# Physical Layer Authentication in Wireless Networks-Based Machine Learning Approaches

**DOI:** 10.3390/s23041814

**Published:** 2023-02-06

**Authors:** Lamia Alhoraibi, Daniyal Alghazzawi, Reemah Alhebshi, Osama Bassam J. Rabie

**Affiliations:** Faculty of Computing and Information Technology, King Abdulaziz University, Jeddah 21589, Saudi Arabia

**Keywords:** physical layer authentication, physical layer security, signal classification, wireless communication, machine learning, deep learning

## Abstract

The physical layer security of wireless networks is becoming increasingly important because of the rapid development of wireless communications and the increasing security threats. In addition, because of the open nature of the wireless channel, authentication is a critical issue in wireless communications. Physical layer authentication (PLA) is based on distinctive features to provide information-theory security and low complexity. However, although many researchers are interested in the PLA and how it might be used to improve wireless security, there is surprisingly little literature on the subject, with no systematic overview of the current state-of-the-art PLA and the main foundations involved. Therefore, this paper aims to determine and systematically compare existing studies in the physical layer authentication. This study showed whether machine learning approaches in physical layer authentication models increased wireless network security performance and demonstrated the latest techniques used in PLA. Moreover, it identified issues and suggested directions for future research. This study is valuable for researchers and security model developers interested in using machine learning (ML) and deep learning (DL) approaches for PLA in wireless communication systems in future research and designs.

## 1. Introduction

With the development of wireless communications and networks, wireless networks have been widely used in various sectors, such as health care, industry, education, and the military, and have become closely related to people’s daily lives. Statista Inc. expects 30.9 billion Internet of Things (IoT) devices to be connected to the internet worldwide by 2025; examples of IoT connections include connected cars, smart home devices, and industrial equipment. In comparison, non-IoT connections—smartphones, laptops, and computers with connections—are predicted to surpass 10 billion units [1]. Furthermore, the various wireless devices’ increased data rates, superior network capacity, and openness in communication carriers make wireless communication and networks more vulnerable to attacks. As a result, security has become a critical issue for the future of wireless networks [2,3].

A secure wireless communication system includes authentication and confidentiality [4,5]. Authentication verifies the user’s identity and prevents adversarial users from accessing the network, while confidentiality guarantees that eavesdroppers are unable to read confidential messages using encryption schemes. Traditional security systems use symmetric and asymmetric cryptographic algorithms to ensure communication confidentiality and authentication. Upper-layer cryptography techniques have been adopted to provide wireless security. Cryptography is a conventional technique to secure a system by utilising the upper layers of the open systems interconnection (OSI) model [3].

Due to the rapid development of ubiquitous computing, traditional security mechanisms, such as cryptographic techniques, have not been efficient in protection. However, traditional security mechanisms face a variety of challenges [6]. For example, the constrained resources of ubiquitous computing devices may not support the computational needs of cryptography authentication techniques that require sufficient resources and high computing ability [7]. Furthermore, with developments in quantum computing, adversaries may be able to execute analytical or brute force attacks, which can be disastrous for any cryptosystem [5]. Likewise, adversaries can easily tamper with traditional authentication mechanisms based on media access control (MAC) or internet protocol (IP) addresses. Consequently, the transformative revolution that ubiquitous computing aims to bring about could be compromised by a lack of secure connectivity. Furthermore, the openness of wireless communication in ubiquitous computing leaves the network unprotected from malicious attacks that imitate legitimate user identities [8]. Therefore, a quick and efficient lightweight authentication method is required to identify unknown wireless transmitters and resolve security threats introduced by adversaries.

Recently, many limited-capacity devices and users have demanded latency-sensitive services that require lightweight authentication techniques [9]. Meanwhile, physical layer security techniques have appeared that provide lightweight security solutions. Physical layer security (PLS) is based on unique physical layer characteristics, including radiofrequency fingerprint (RFF), wireless channel state information (CSI), receive signal strength (RSS), and channel impulse response (CIR), to provide security services. As a result, the physical layer characteristics of wireless channels and hardware have become unique and consistent, which are hard to alter by users and can be used to identify wireless transmitters.

Machine learning (ML) is a subset of artificial intelligence (AI) that emerged from pattern recognition [10]. Lately, research in wireless communication has noted the distinction and effectiveness of machine learning by identifying the probability of learning based on signal classification [11] and specific emitter identification [12,13]. However, ML algorithms may face difficulty handling high-dimensional data because of the sizeable signals of raw data. Innovations in ML delivered a new learning technique called deep learning (DL) [10]. Deep learning has improved image and speech recognition by solving complicated multiclass classification problems [14]. Deep learning is a machine learning algorithm that models the function of increasing complexity by adding more layers and more nonlinear processing neurons within a layer; it can learn higher-level representations of input data [15]. Therefore, deep learning is a suitable model for this problem because it can learn from data from different wireless signals. With the evolution of deep learning in recent years, researchers have begun to use DL techniques for classifying a wireless signal in terms of signal recognition [16,17,18,19] and modulation scheme classification [20,21,22].

In general, one can hardly guarantee the secure communications of many ubiquitous computing devices with limited wireless resources and challenging issues in developing security techniques. Ubiquitous computing is located in the heterogeneous networks nearest the users and adversaries who can easily capture the terminals and launch malicious activities. Secure authentication in ubiquitous computing is also complicated by the promiscuous nature of the wireless transmission medium and the limited hardware and software capabilities of the nodes in such networks. Furthermore, most ubiquitous computing devices have limited resources, computation, and power. Accordingly, it is impossible to perform data caching and follow traditional cryptography-based security computations. As a result, a lightweight and secure authentication technique needs to be proposed. By taking advantage of the physical layer characteristics of transmission media, physical layer authentication (PLA) can execute lightweight authentication as well as successfully manage the trade-off between the security and low-latency requirements of the wireless nodes compared with the upper-layer authentication mechanisms [23].

To the best of our knowledge, no previous study has conducted a systematic literature review (SLR) on physical layer authentication in wireless network-based machine learning approaches, which makes it hard to determine the maturity and proficiency of physical layer authentication techniques to solve wireless networks’ security challenges. Accordingly, this paper conducts a systematic review to identify, analyse, and evaluate existing PLA solutions used to secure wireless communication and networks. To this end, we aim to answer the following research questions:

- What machine learning approaches are applied in physical layer authentication to secure a wireless network?
- What are the existing physical layer authentication techniques for handling a wireless network’s security problems?
- What are the existing key challenges, open issues, and future trends in wireless network security based on physical layer authentication solutions?

To conduct this SLR, we followed the methods introduced by Kitchenham [24] to provide a clear vision of the existing research and highlight new research guidelines. This SLR has resulted in constructing and delivering a knowledge base of current PLA techniques to secure the context of wireless networks for practitioners seeking to comprehend the current techniques and methods applied and researchers aiming to explore existing gaps. The main contributions of this paper are as follows:

- Present a systematic review of the current state of the art in physical layer authentication based on machine learning and deep learning approaches.
- Provide an assessment of ML and DL algorithms used for physical layer authentication.
- Review the methods of physical layer authentication and compare their performance.
- Outline the primary challenges and issues confronting physical layer authentication techniques.
- Define the key aspect in which future research can improve the use of PLA for wireless network security.

The remainder of the paper comprises five sections. Section 2 explains the related reviews and surveys in physical layer authentication, and Section 3 describes the background. Section 4 presents the research methodology, research questions, scope, and SLR process, and Section 5 discusses the analysis of the results. Finally, Section 6 draws conclusions and identifies areas for further research.

## 2. Related Works and Motivation

This section explores previous literature related to physical layer authentication techniques to emphasize the need for this SLR. Great review studies published in physical layer security are summarized in Table 1. Next, surveys related to PLS, and PLA are discussed in Section 2.1 to illustrate the lack of comprehensive reviews and to indicate the benefits and weaknesses. Then, based on a systematic search, the motivations for conducting this investigation are declared in Section 2.2.

### 2.1. Related Review Studies on Physical Layer Security and Authentication

Shu et al. [25] explored security issues related to the physical layer in cognitive radio networks. They also gave an overview of several existing security attacks and surveyed the related countermeasures on how to defend against these attacks. However, the study did not propose an SLR, and the number of papers included in the literary survey was only eight. Liu et al. [4] provided a comprehensive overview of the fundamentals and technologies of PLS. Further, they discussed the challenges and solutions in different PLS technologies, ranging from wiretap coding, secure signal processing, and physical layer key generation to physical layer authentication. In this survey, sources published before 2016 and recently published papers were not mentioned.

While Wu et al. [2] investigated research subjects on the system designs of physical layer security and reviewed deep insights into performance metrics and fundamental optimization problems, the emerging development of future wireless technologies has brought new security challenges for 5G networks. Wang et al. [3] and Sánchez et al. [26] provided surveys summarizing the latest research results on physical layer security for several promising 5G technologies, including heterogeneous networks (HetNet), massive multiple-input multiple-output (MIMO), non-orthogonal multiple access, physical layer security coding, millimetre-wave (mm-wave) communications, and full-duplex (FD) technology, and the fundamental concepts of each technology. As the number of IoT devices expands and new industries utilize IoT technologies, the need for IoT security will rise. Thus, Rojas et al. [27] explored some IoT communication protocols and recent developments in PLS techniques and solutions that use beamforming, spread spectrum coding, and radio frequency (RF) fingerprinting. However, since the research approach was not systematic, the method of selecting research papers and their time range are unidentified.

Multiple survey papers point to physical layer authentication as a promising technique in wireless security communications. Bai et al. [28] provided a comprehensive review of the domain of PLA in wireless communication systems, including the concepts and frameworks of key-based/less PLA systems and common attack models. In addition, Bai et al. discussed key techniques applied in PLA systems. Xie et al. [29] provided an exhaustive survey of features and techniques used in PLA, categorizing existing PLA schemes into passive and active and presenting the significant differences between the two categories. The research was recently published, but a systematic approach had not been considered. Recently, machine learning has appeared as a promising tool for reducing the increasing complexity of wireless networks. The integration of ML and PLS has also enticed some research interest. Kamboj et al. [10] reviewed physical layer authentication, antenna selection, relay node selection, and integration with ML, and Jiang [30] presented reviews of PLA schemes using machine learning for the 5G-based IoT. They also compared PLA schemes. Aside from comparisons between PLA schemes, they examined machine learning techniques used in intrusion detection, access control, and anomaly detection to provide other security advantages in the 5G-based IoT. Angueira et al. [31] provided a comprehensive review of the security challenges of radio-frequency wireless systems in industrial. Angueira et al. also presented physical layer attack families and countermeasures, including a discussion on the effectiveness of the described solutions.

### 2.2. Motivations for a Secondary Study on PLA in Wireless Network Security

Physical layer authentication plays a considerable role in wireless security communications. Before performing this review, we came across different papers applying PLS and PLA to wireless networks for security purposes. As shown in Table 1, we looked at several factors, including year of publication, publisher, main topics, inclusion of ML, environment, and focused issues. Obviously, no SLRs had collected only works relating to PLA. Although the studies mentioned dealt with PLS, few focused on PLA, and none focused on deep learning techniques. Nevertheless, we encountered reviews [28,30] directly related to PLA and machine learning.

Consequently, the main contribution of this study is filling a gap in the required detail and analysis of potential physical layer authentication systems based on ML and DL techniques for various security purposes. In addition, we aim to present brief and valuable content for newcomers to catch up in these areas. To the best of our knowledge, no previous surveys have classified and analysed PLA studies that used deep learning technologies to improve security service performance. The study discusses the challenges and description of the most remarkable issues and future trends of PLA in wireless networks and communication.

Recently, physical layer authentication methods have attracted substantial research interest. Unfortunately, none of the retrieved secondary studies in Table 1 addressed our research questions detailed in Section 4.1. Therefore, insights into identifying open issues and guidelines for future research are provided. This work examines research studies published since 2015 on PLA systems intended to secure wireless networks. Through the paper selection process, 248 papers were chosen and narrowed down to 31 primary studies. This survey will directly help academics and professionals understand PLA developments to secure emerging wireless technologies.

## 3. Background

In this section, we briefly explain the physical layer in a wireless network, followed by a brief description of PLS and PLA. Finally, the signal classification subject is presented.

### 3.1. Wireless Network

**Figure 1:**
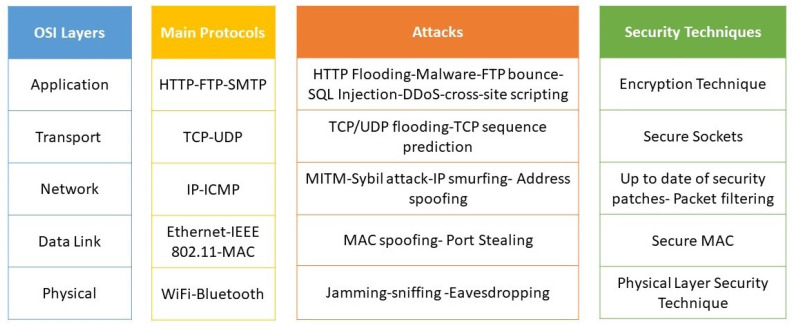
A generic wireless OSI Model information consisting of the layers, main protocols, main attacks, security techniques.

A network’s architecture defines the protocols and components required to meet application needs. The OSI Model practically represents a wireless network’s different standards and compatibility. The OSI model is a conceptual framework that outlines how data are exchanged within a computer network from one device to another. The OSI model describes a complete set of network services within each network component organized into layers, illustrated in Figure 1. Each layer consists of a collection of conventional communication protocols and customized components to accomplish specific functions.

#### 3.1.1. Physical Layer

The physical layer is the only layer in the OSI model interacts with actual hardware, transmission, and signaling mechanisms. The physical layer transmits raw bits over a physical data link connecting network nodes by converting them to electrical pulses, representing the binary data. The electric pulses are then converted to electromagnetic waves to be transmitted wirelessly. On the other hand, the physical layer specifies the data transmission mechanism and how data can move between devices.

#### 3.1.2. Wireless Physical Layer Protocols

Recently, massive of advanced wireless technologies and dozens of different wireless protocols meet the needs, each with its performance characteristics and optimized for a specific task and context. However, various wireless protocols exist, such as WiFi, Bluetooth, ZigBee, NFC, WiMAX, LoRa, 5G, satellite services, and more. Therefore, it is necessary to be aware of the system’s constraints and performance requirements when choosing protocols. Power, data rate, reliability, and range are essential metrics for distinguishing between protocols [27].

#### 3.1.3. Wireless Networks Physical Layer Threats

The openness of wireless networks produces communication more vulnerable to attacks, which poses severe challenges for network security. Wireless networks have security vulnerabilities, such as [4,6,27,32]:

Eavesdropping: Unauthorized and unannounced interception of communications between devices. Through eavesdropping, the intercepted messages can be exploited for future illegal purposes. Eavesdropping attackers can be categorized as active eavesdroppers and silent eavesdroppers. The distinction is that active eavesdroppers acting as communication parties unintentionally send signals to transmitters, which CSIs can extract through estimation. On the other hand, silent eavesdroppers snoop on messages while being silent, where their CSIs are not available for transmitters. Therefore, this kind of threat can be divided into two types based on the manner of the attacker: interception and traffic analysis.

- Interception: Eavesdropping is the most common attack on wireless devices’ privacy. The attacker could find legitimate communication by snooping in the nearby wireless environment when the traffic transmits control information about the sensor network configuration.
- Traffic Analysis: The ability to track communication patterns to facilitate various types of attacks.

Jamming: Blocks legitimate communications between devices by saturating a channel with noise, which can direct denial-of-service (DOS) attacks at the physical layer. In general, jamming attacks can be divided into proactive and reactive jamming.

- Proactive Jamming: Proactive jamming attackers spread interfering signals whether the legitimate signal communication is there or not. To save energy and toggle between the sleep and jamming phases, attackers sporadically spread random bits or normal packets into networks. Attackers sporadically broadcast either random bits or conventional packets into networks to preserve energy and rotation between the sleep and jamming phases.
- Reactive Jamming: Attackers that use reactive jamming can monitor the legitimate channel’s activity. If there is an activity, the attacker transmits a random signal to interfere with the existing signal on the channel.

Contaminating: Attackers seek to contaminate the channel estimate phase to gain unfair advantages in the communication phase that follows. In the same context, a feedback contamination attack means that the attacker can use falsified feedback to force the transmitter to command their beams to attackers different than the intended users.

Spoofing: Attackers try to enter or corrupt legitimate communications by transmitting a deceiving signal with a higher power in the transmission phase between transceivers or monitoring the legitimate transmitter for sending a falsified signal between two legitimate signals. This kind of attack has different implications, such as the intrusion of an adversary into the local network or injecting some falsified identity information. There are two types of spoofing attacks: identity spoofing attacks and Sybil attacks.

### 3.2. Physical Layer Security

The world has become increasingly online and connected via wireless networks recently. Additionally, wireless devices are increasingly employed in a variety of sectors. For example, smart things, mobile communication, unmanned platforms, drone control, autonomous driving, etc. Unlike wired networks, the openness of the wireless network allows nearly all wireless receiving devices within their range to receive signals [33,34]. This feature gives legal and illegal users the same access to the communication channel. However, protecting the integrity, confidentiality, and availability is challenging in wireless networks [33].

Information security mainly depends on cryptographic techniques to achieve communication security requirements, including authenticity, confidentiality, integrity, and availability [2,4,35]. Authenticity verifies communicating entities. Data integrity validates that transmitted data are not changed. Data confidentiality assures that transmitted data did not expose to unauthorized entities. Finally, data availability prevents adversaries from interrupting access to data.

Using encryption-based security technologies at application layers has enhanced wireless security. Still, their inherent vulnerabilities are heavy computation and key management, resulting in high complexity and resource consumption [2,35,36]. Cryptographic techniques have efficiently protected modern communication and computer networks. However, it is not entirely suited to the future of ubiquitous computing, which will be elaborated on in the following.

Traditional cryptographic approaches are computationally secure because the attacker cannot decipher the protection within a specific time. However, it may be compromised due to the progress in quantum computing advances. However, because of advances in quantum computing, it may be compromised. For example, the quantum search algorithms such as Grover’s and Shor’s algorithms exploited the discrete logarithm problem that current cryptographic mechanisms heavily rely on [37]. Traditional authentication techniques are based on the IP or MAC addresses as the identity, which can be easily tampered with by malware attackers [38,39]. In addition, cryptographic algorithms rely heavily on computational complexity and secret keys [5,28]. As a result, these algorithms perform effectively on devices with high processing capabilities, like smartphones. In comparison, many IoT devices are low cost and small, equipped with limited storage memory, and powered with batteries, making it impractical to implement complicated cryptography-based security protocols.

Shannon first considered the confidentiality of PLS was assumed in 1949 and proposed the first application of information theory to cryptology, also known as Shannon’s information-theoretic secrecy [40]. Then, approximately three decades later, one of the most targeted studies the physical layer confidentiality is to maximize the secret information rate received by the legitimate user in the wiretap channel, which is defined as the secrecy capacity by Wyner [41]. Wyner’s work set the basis and inspired PLS research, with scholars proposing various PLS techniques for different purposes.

Wireless network security was previously thought to be a high-layer problem that could be handled with cryptographic approaches [42]. The situation changed in the first decade of the 21st century when wireless networks started to spread around [37]. Therefore, physical layer security based on information theory has appeared as a promising approach to protecting wireless communications to achieve information-theoretic security against eavesdropping attacks, for instance. Compared to cryptographic techniques executed at upper layers, physical layer security offers two significant advantages:

- First, physical layer security techniques do not rely on computational complexity compared to cryptography techniques [3,4,26,38,43]. As a result, the achieved level of security will not be compromised; even if the unauthorized devices in the wireless network are provided with powerful computational capabilities, secure and safe communications can still be performed.
- Second, physical layer security techniques have high scalability [3,43]. Wireless devices always join or exit the network at any time; due to the decentralized nature of the network, the PLS technique can provide secure data communication in the network.

### 3.3. Physical Layer Authentication

The inherent broadcast nature of wireless communications raises security and privacy issues where adversaries can launch different types of attacks. Accordingly, authentication is an important issue in wireless communications [29]. Device identity authentication requires safeguarding wireless networks to validate whether the users are legitimate and allowing them to access the network while preventing malicious users [39]. Most existing wireless communication systems perform authentication through upper-layer authentication techniques that are typically implemented using cryptography-based authentication algorithms [39]. However, traditional authentication approaches depend on software addresses such as IP and MAC addresses, which can be tampered with or forged [44]. Once adversaries obtain the security credentials, they can pretend as legitimate users to reach private data and launch severe attacks on the wireless devices [44,45].

However, upper-layer authentication mechanisms based on traditional cryptography-based algorithms are unsuitable for advanced wireless communication systems [29]. For example, cognitive radio networks, IoT, internet of vehicles (IoV), smart grids networks, and unmanned aerial vehicles (UAV) because of the following issues [6,29,39,46]: With the advancement in computational power and cryptanalysis algorithms, the time it takes to crack a cryptography key has been drastically reduced. However, because the upper layer signaling is not altered, the replayed signal can successfully spoof the legitimate receiver. Therefore, the complicated cryptography techniques in upper-layer operations, e.g., encryption, decryption, and frequent authentication handovers, are unsuitable with limited capability for wireless devices. Furthermore, the process of key sharing and management introduces overhead concerns in massive ubiquitous computing scenarios, such as the amount of storing excessive keys or defending against the eavesdropping attacks of frequent exchanging keys.

**Figure 2:**
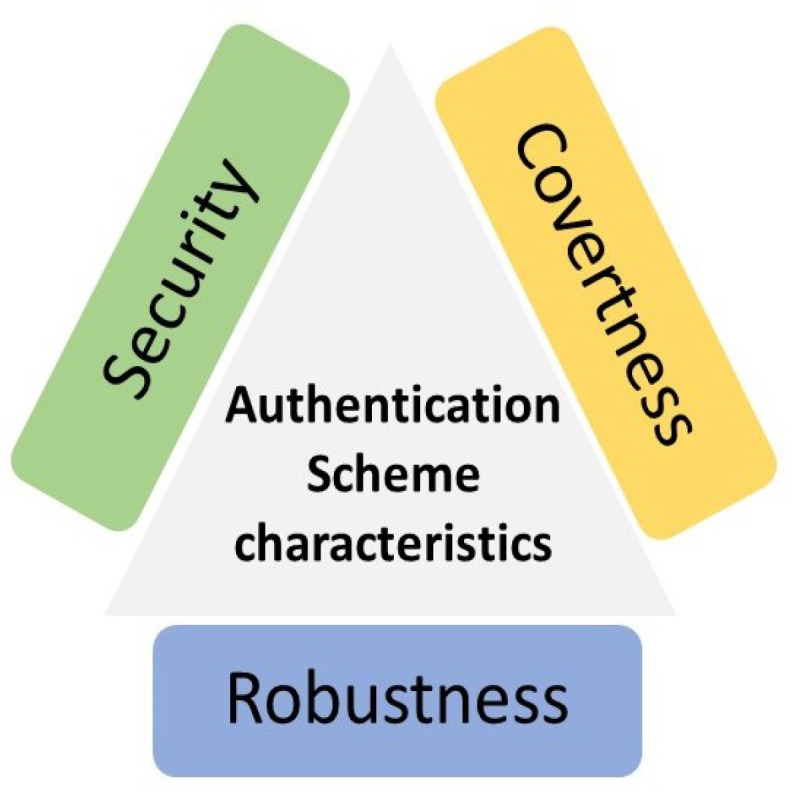
Authentication Scheme Characteristics.

Wireless physical layer authentication is a method of validating a wireless transmitter by checking the physical layer characteristics of the communication [46]. A good authentication scheme should generally have three characteristics: covertness, robustness, and security [28], as demonstrated in Figure 2.

- The covertness means that any authentication schemes should not significantly affect the performance of the standard data transmission, do not occupy too much communication overheads or extra computational resources, and do not harm the existing conventional higher-layer cryptographic-based techniques.
- Robustness requires that the PLA framework is robust enough to mitigate channel fading and noise interference.
- Security is the kernel of PLA systems, representing the ability to prevent the authentication procedure from being interrupted or invaded by eavesdroppers.

Recently, PLA has attracted much research interest compared to traditional secret key-based authentication techniques because of the following advantages [29,46]:

- The PLA allows a legitimate receiver to easily distinguish between a legitimate and adversary transmitter without upper-layer processing, decreasing computational complexity and processing delay.
- There is no key distribution and management need with PLA compared to conventional secret key-based authentication schemes. Instead, some existing physical layer authentication approaches rely on analog channel information and device-specific characteristics caused by manufacturing variability.
- In a heterogeneous coexistence system, incompatible devices may not be able to decode each other’s upper-layer signaling, but they should be able to decode the physical layer bit-streams.
- The PLA presents information-theoretic security, where the physical layer puts adversaries in a state of uncertainty.

#### Physical Layer Authentication Techniques

The mostly studied authentication techniques can be classified into: radio frequency fingerprint-based and channel-based schemes.

**Physical Layer Authentication based on Radio Frequency Fingerprint**

Toonstra et al. [47] first proposed the concept of "radio frequency fingerprint" technology in 1995. radio frequency fingerprint is similar to human fingerprint biometric identifiers, but they are extracted from wireless signals [48]. Therefore, the RFF can identify and classify wireless devices as an advanced technique for wireless security [39]. In addition, RF fingerprinting can provide a novel approach for emitter identification using the external signal feature rather than the information content [11]. Radio frequency fingerprinting was created from the imperfections in components of a wireless device raised during the production process, which is a small feature reflected in the launching signal [44,45,49]. These imperfections deviate slightly from their nominal specifications and thus do not impact normal communication functions, allowing device identifiers to be obtained from the component’s imperfections. Since the RFF of the wireless device defines unique characteristics that are very difficult to manipulate and forge [50].

Radio frequency fingerprint identification (RFFI) is a potential wireless device authentication technique that uses hardware fingerprints to identify wireless devices [44,45]. HALL et al. [51] proposed the concept of radio fingerprint identification in the wireless network device identification field. Because most IoT end nodes have limited computational and energy resources, the RFFI approach does not impose any additional power consumption on the devices to be authenticated. Consequently, RFFI is particularly suitable for low-cost wireless devices such as IoT [45]. The RF fingerprint-based identification comprises two phases: training and classification [39]. During the training phase, the receiver will first sample received signals from the devices under good channel quality, extract features, then save them as a reference template. In the classification phase, the receiver will acquire signals from prospect devices, compare the same type features with the reference template, and classify the devices based on similarity.

**Channel Based Authentication**

Since the wireless channel has the characteristics of space-variability, uniqueness, time-variation, and reciprocity, the communication channels between the transmitter and the receiver in different places are different. The physical layer characteristics verify the uniqueness of wireless channels on the communication parties. The physical layer authentication based on wireless channels uses the channel diversity generated by spatial variability to achieve authentication.

The PLA techniques can identify the legitimate and illegal nodes by examining channels characteristics, such as received signal strength (RSS), channel impulse response (CIR), channel state information (CSI), and channel frequency response (CFR). RSS symbolizes the strength of the received signal. On the other hand, the CIR is a practical tool for designing and implementing communications systems because it shows how the waveform changes as it transits through the environment [52]. Moreover, it captures the reflection, absorption, diffraction, delay, and attenuation. Furthermore, the CSI represents the channel feature of a communication link [53]. CSI describes characteristics and effects of, e.g., scattering, fading, and power decay on the wireless signal propagation from the transmitter to the receiver at specific carrier frequencies [54,55,56]. However, due to scattering and reflection, the CSI is difficult to predict and emulate. The wireless channel’s uniqueness in time and space lets it map different places with spatial and temporal environment characteristics [8]. In the context of channel-based authentication schemes, both RSS and CIR show unique spatial properties due to path loss and multi-path effects [57]. Compared to physical layer features that reflect large-scale fading in the channel, CSI includes location information details and represents the deeper channel differences.

### 3.4. Signal Classification

With the evolution of wireless communication technology, signal recognition and classification have become necessary. Defined signal intelligence is a field of study and application that relies on extracting signal characteristics such as protocols, bandwidth, center frequency, modulation, and emitter identity from unknown radio frequency signals [58]. This field of study is further divided into subcategories dependent on the task. Automatic modulation classification and specific emitter identification are the most well-researched studies.

#### 3.4.1. Automatic Modulation Classification

Automatic modulation classification (AMC) classifies radio signals by identifying modulation types, schemes, and types of wireless transmission [20,59,60]. AMC can extract digital baseband information even under limited prior knowledge [59]. Moreover, AMC is important for many applications, including signal detection, spectrum monitoring, software-defined elastic optical networks, and cognitive radio [61,62]. To achieve efficient transmission, transmitted signals are generally modulated using different modulation techniques to encode data on multiple carriers frequencies. The most well-known modulation methods are 4FSK, 16QAM, BPSK, QPSK, and OFDM. The AMC method can be categorized as likelihood-based and feature-based approaches [21,22,63], where likelihood-based classifiers need parameter estimation. On the other hand, feature-based approaches do not require parameter estimates and have recently increased interest. In contrast, feature-based approaches consist of two steps: feature extraction and classification, which can make decisions based on a specific criterion.

#### 3.4.2. Specific Emitter Identification

Specific emitter identification (SEI) is a method for identifying individual emitters by signal features extraction and analysis [64,65]. The SEI defines the individual emitter by distinguishing it from a group of emitters by obtaining emitter operating parameters and performance information by comparing the characteristic parameters of signals intercepted by receivers. The SEI is often utilized in intrusion detection systems to improve the security of radar, satellite communication, and radio frequency systems. The SEI procedure generally comprises four steps [66,67]: signal acquisition and preprocessing, extraction of features from the transmitted signal, and classification recognition by matching the features with database reference, assigning the best matching aggregate to these features. The signal acquisition and preprocessing step eliminates uncertain factors that affect the signal. To obtain better identification performance, extracting features that can effectively reflect the subtle differences between devices is necessary. Existing feature extraction methods involve transient and steady features. For identification, current state-of-the-art SEI systems rely on measuring pre-determined and expert-defined signal features clustered by emitter [68]. However, expert-defined signal features require a lot of the raw signal data to be preprocessed, for example, synchronization, carrier tracking, demodulation, signal-to-noise ratio (SNR) estimation, and the computational cost of measuring or estimating the expert features [67]. Moreover, their different domains extract features in signal processing, including the time domain, frequency domain, and time-frequency domain [13]. As well, these domains are based on five signal parameters pulse repetition interval (PRI), the direction of arrival (DOA), pulse frequency (PF), pulse width (PW), pulse amplitude (PA); all these parameters are combined in a pulse descriptive word (PWD) [16,69].

## 4. Research Methodology

The survey conducted in this paper is based on systematic literature review methodology. A systematic literature review (SLR) is a form of secondary study that uses a well-defined methodology to identify, evaluate, and interpret all available research related to a specific area or research questions in an unbiased and repeatable way [24]. However, we split our methodology into two phases. The first phase was to determine the research questions based on motivation. The second phase involved defining the research strategy for finding relevant research publications, explaining specific search terms, and the appropriate paper selection criteria. We also defined the quality assessment rules used to refine the results. The following subsections describe the rules that were followed in this study.

### 4.1. Phase 1. Planning the Review

Planning begins with identifying the need for an SLR method, defining the research questions that the systematic review will address, and producing a review protocol defining the basic review procedures.

Step 1. Identifying the motivations: The motivation is addressed, and the contribution of this systematic review is described in Section 2.2.

Step 2. Defining the research questions: Defining the research questions is essential. By answering the research questions, literature reviews reach their goals. Therefore, the research study questions for this systematic review are shown in Table 2.

Step 3. Designing a review protocol: Reducing the possibility of bias among researchers is the principal aim of this pilot study. Consequently, we considered the research questions to support the review protocol. We followed the integration search method, which includes comprehensive automated searches in different online resources, and a manual review of the selected papers. In addition, the set of inclusion/exclusion criteria was improved.

### 4.2. Phase 2. Conducting the Review

**Figure 3:**
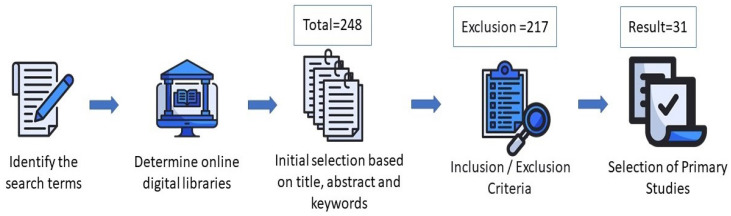
Primary study selection process.

In this subsection, we describe each step in choosing papers and the search process based on SLR methods, as depicted in Figure 3. This approach to identifying relevant papers has four steps.

**Step 1. Selecting of primary studies:**

This step aims to find as many papers related to our research as possible. Then, we will determine which sources to search for and identify the search terms to be applied.

*Identify the Search Terms*: We defined the search terms used for the manual searches in online scientific databases. A general approach is to break down the research questions into individual terms and then make a list of synonyms and abbreviations. We divided the key terms into three categories, each of which included synonyms or variations of the terms relevant to the research question:

- *Group 1 finds papers related to transmitter identification*: (“Transmitter Identification” OR “Transmitter Classification” OR “Specific Emitter Identification” OR “Emitter Classification”) AND (“Deep Learning” OR “Neural Network” OR “Radio Frequency Fingerprints” OR “RF fingerprinting” OR “RFF”).
- *Group 2 finds papers related to wireless network security*: (“Wireless Networks Security” OR “Wireless Device Security” OR “Internet of Things Security”) AND (“Transmitter Identification” OR “Transmitter Classification” OR “Specific Emitter Identification” OR “Emitter Classification”).
- *Group 3 finds papers related to physical layer authentication*: (“Physical Layer Authentication” OR “Physical Layer Security”) AND (“Wireless Networks” OR “Wireless Device” OR “Internet of Things” OR “IoT” OR “Radio Frequency Fingerprints” OR “RF fingerprinting” OR “RFF”).

**Step 2. Specifying the Studies’ Sources:**

**Figure 4:**
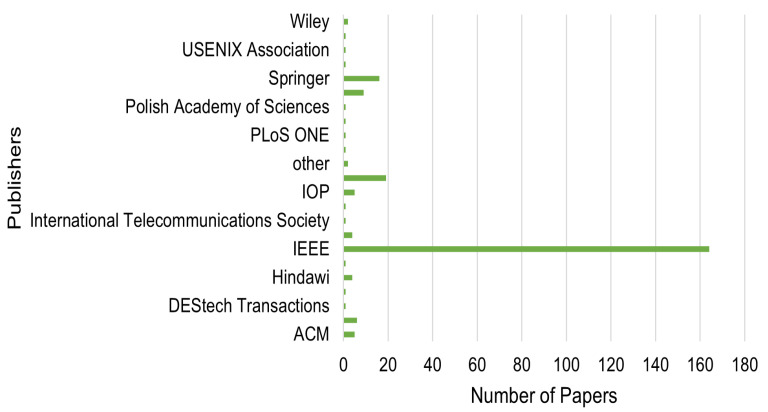
Distribution of papers by publisher.

After identifying the search keywords, we determined which online digital libraries and journals to search. We initially searched for related studies on the online platform Google Scholar, based on the search keywords chosen in the first stage. Later, we included more related articles by searching in recognized academic publishers such as IEEE Xplore, ACM Digital Library, Science Direct, Springer, and Hindawi. Conducting the selected studies by database source is depicted in Figure 4.

**Step 3. Selection of Primary Studies:**

When potentially relevant primary studies have been obtained, they must be assessed for their actual relevance and whether they may answer the research questions. Selection of primary studies is considered a multistage process.

**Figure 5:**
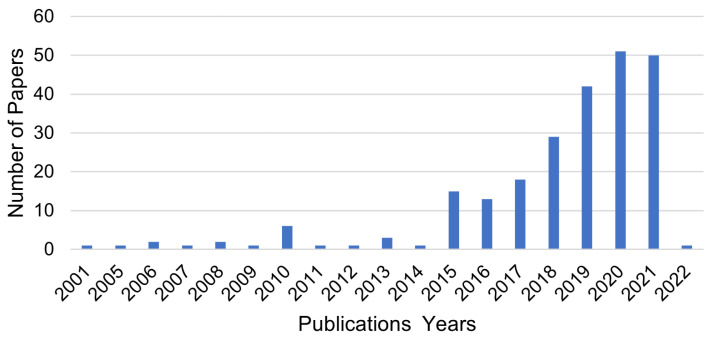
Distribution of publications by year.

**Figure 6:**
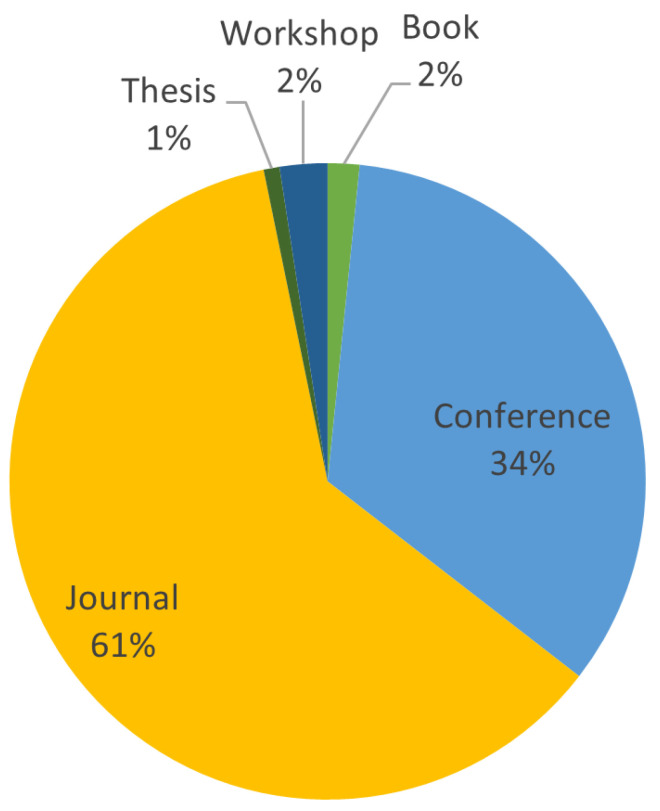
Distribution by type.

**Stage 1:** This stage involves filtering potential primary papers by scanning their titles, abstracts, and keywords. This stage yielded 248 papers, including journals, chapters, conference papers, books, and other publications that responded to the search terms. The total number of selected publications is shown in Figure 5, and Figure 6 indicates the type of selected publications.

**Stage 2:** In this stage, to restrict the number of studies for review, we adopted inclusion and exclusion criteria, as mentioned in Table 3.

**Stage 3:** We eliminated duplicate studies from the set of 248 papers.

**Step 4. Study Quality Assessment:**

An essential part of writing an SLR paper is choosing high-quality literature to make the most accurate and reliable review. Quality assessment rules (QARs) were applied using a group of research questions to assess the quality of studies. Ten QARs were classified, each worth one point out of 10. The rate of each QAR was selected as follows: completely answered = 1, above-average answer rate = 0.75, average answer rate = 0.5, below-average answer rate = 0.25, completely unanswered = 0. If the summation of all ten QARs was less than 5, the study was excluded.

The quality assessment rules used to assess the quality of the studies in Table 4 were as follows:

QA1: Are the research objectives clearly explained?

QA2: Is the study set in the context of other studies and research?

QA3: Is the specific field of wireless network security clearly defined?

QA4: Is the proposed system model clearly identified and acceptable?

QA5: Are the ML/DL techniques appropriately described?

QA6: Is the design of the experiment appropriate and adequate?

QA7: Was the study executed on a sufficient dataset?

QA8: Is the implemented experiment appropriately described?

QA9: Are the results of the experiments well justified?

QA10: Do the results support the findings presented?

The scoring results from applying the QARs are shown in Table 5.

## 5. Analysis

Considering the process described in Section 4, this section analyses the results of the SLR method. According to the research questions defined in Section 4.1, we respond to RQ1, RQ2, and RQ3, as indicated in Table 2. In addition, these research questions are discussed in separate Section 5.1, Section 5.2 and Section 5.3.

### 5.1. RQ1: What Machine Learning Approaches Are Applied in Physical Layer Authentication to Secure Wireless Networks?

**Figure 7:**
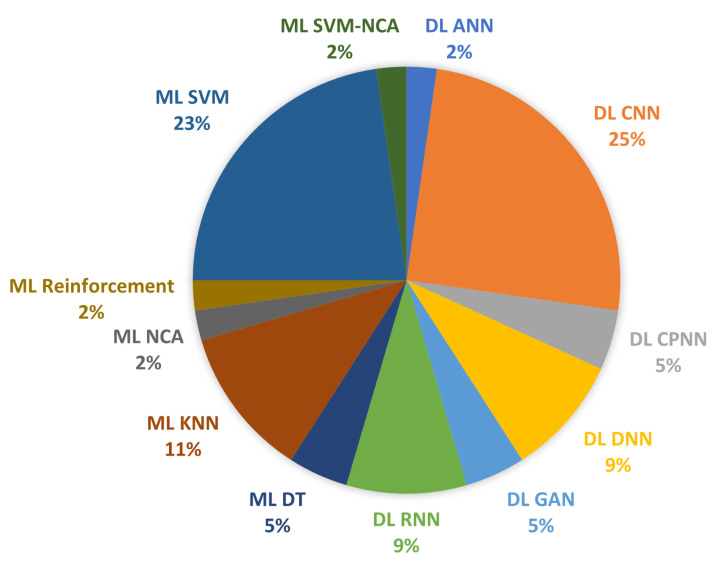
Distribution by type.

**Figure 8:**
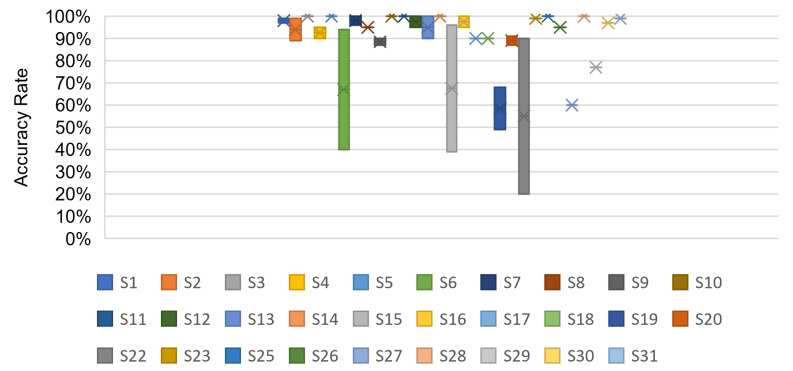
The performance results of selected studies.

As artificial intelligence technologies advance, machine learning and deep learning are progressively expanding in all parts of people’s lives. Recently, advances in ML and DL have enhanced the reliability of classification in physical layer authentication. Therefore, we present various learning approaches for physical layer authentication to secure wireless networks and discuss the algorithms utilised. Figure 7 represents the percentage of ML and DL algorithms in the reviewed papers. In the research papers, we observed 12 algorithm types; 45% of the studies applied ML algorithms, and 55% applied DL algorithms to implement their solution. In addition, we compared the chosen studies, as shown in Table 6. This comparison reflects approaches, algorithms, evaluation types, features, performance metrics, tools, and platforms. The performance results of selected studies are shown in Figure 8.

#### 5.1.1. Machine Learning Based PLA

Machine learning is a subset of artificial intelligence that emerged from pattern recognition. The first definition of machine learning was provided six decades ago by Arthur Samuel [97]. Machine learning approaches exploit past data for model generation and adapt to their conditions through experience. These developed models can generate outputs from given inputs without human intervention. The ML algorithm can observe the relationship between input and output because of the large amount of data available and repetitive interactions.

Some studies presented a classification method based on support vector machine (SVM). The SVM algorithm has great theoretical and practical advantages when handling difficulties like small samples, nonlinearity, and high dimensions. Study **(S6)** proposed a classification method based on SVM to identify cloning RF devices and deal with binary classification problems. However, the study compared classification models one based on traditional logistic regression (LR) and another based on SVM. The authors found that the accuracy of LR is not as good as SVM. In the same context, S11 developed a model using RF-distinct native attribute fingerprints and an SVM algorithm for device verification and rogue device rejection. Furthermore, **(S25)** proposed a channel authentication method based on an SVM algorithm to enhance the performance of the authentication detection method and decrease the hardware’s complexity.

To aid the identification models in multiple wireless transceiver scenarios, **(S13)** presented a model based on SVM to identify multiple wireless transceivers. In addition, **(S27)** suggested a lightweight multiuser PLA mechanism to prevent illegal nodes from falsifying the identity information of legitimate nodes to access industrial wireless networks. The mechanism is based on SVM, which utilizes down-sampling and parameter optimization to reduce computational complexity and solve the problem of low authentication accuracy. To obtain the best multi-classification, three optimization algorithms were applied a grid search algorithm, a particle swarm optimization algorithm, and a genetic algorithm to search for the best parameter pair.

The study **(S8)** proposed an integrated support vector data description (SVDD) model to solve identity authentication problems in the mobile IoT. SVDD is established based on statistical learning theory, inherits its advantages, develops continuously, and has a complete theoretical foundation and basis. The study also used the whale swarm optimization algorithm (WOA) to optimize the parameters in the SVDD model. This algorithm has the advantages of fewer adjustment parameters, simple operation, and strong local optimal ability. Moreover, **(S23)** presented an SVM classifier combined with a neighbourhood components analysis (NCA) algorithm for reliable feature selection. The NCA placed the same label of feature components close to each other, and the cases of different labels were placed as far away as possible in the feature space.

To detect adversary attacks, **(S18)** developed two models to detect clone and Sybil attacks using CSI. The authors utilised SVM as an attack detection algorithm for two main reasons. First, attack detection is a two-class problem with only two results. Second, the SVM algorithm probably achieves better classification results with small offline training sample sets. Furthermore, to detect an eavesdropper during the authentication phase, **(S30)** applied sophisticated SVM classifiers, in which they constructed two models, depending on whether the channel state information (CSI) was known or unknown.

**(S20)** developed an intelligent decision method based on the AdaBoost learning algorithm to detect spoofing attacks. AdaBoost is an acronym of ‘adaptive boosting’ developed by Yoav and Robert [98] and is the most extensively used form of boosting algorithm. AdaBoost is a powerful technique that can be used in conjunction with base classifiers to create a committee form that outperforms other base classifiers. The AdaBoost principle states that it improves its performance through an adaptive iterative process in prospective weak classifiers, known as learners, that are altered to enhance cases misclassified by prior classifiers.

Due to the limited computing ability and high real-time requirements of IoT devices, the identification algorithm must meet the computational requirements. Therefore, in **(S4)**, a method was proposed for recognizing individual industrial internet of things (IIoT) devices, preventing and detecting MITM attacks in the IIoT environment by using a k-nearest neighbours (KNN) classifier.

In addition to the above, the researchers in **(S21)** suggested two models for spoofing detection in dynamic wireless networks by leveraging the channel states of radio packets, which establish the test threshold based on reinforcement learning without knowing all channel parameters. The first model is based on Q-learning as a simple reinforcement learning technique; Q-learning allows each classifier to learn its optimal strategy in dynamic environments. For example, in spoofing detection with Q-learning, the receiver builds the hypothesis test to determine the sender for each packet received in the time slot. The second model is based on Dyna-Q as an extension of Q-learning; Dyna-Q uses Dyna architecture to formulate a learned-world model that consists of the significant functions of the online planning receiver. Dyna-Q accelerates learning in a dynamic environment with unknown parameters by obtaining hypothetical experiences from the world model. As a result, spoofing detection with Dyna-Q improves performance over Q-learning.

#### 5.1.2. Deep Learning Based PLA

In recent years, the deep learning computing approach has been termed the Gold Standard in the machine learning community [99]. It has gradually become the most extensively used computational approach in machine learning, generating exceptional results for various complex cognitive tasks. More importantly, DL has outperformed well-known ML techniques in many domains because of its powerful data analysis ability and has proved to be robust, dependable, and accurate. As a result, DL has recently been increasingly combined with physical layer authentication and secure wireless communication systems.

Various studies selected convolutional neural networks (CNN) for their reliability and powerful learning features with high accuracy and low loss function in the training process. In **(S7)**, three classification approaches were applied for the physical layer authentication problem, based on a combination of CNN and recurrence plots (RP) techniques. The recurrence plots technique is considered a visualization tool that aims to explore a multidimensional phase space trajectory through a 2D representation of its recurrences. The RP technique was used to visualize the recurrences of dynamic systems in the context of PLA and transform the digitized RF emissions before submitting them to a CNN classifier. To secure wireless communication in the WLAN system, **(S10)** proposed a methodology based on CNN to identify different devices by taking advantage of unique RF fingerprints.

For multi-user authentication, **(S17)** proposed a CNN model to distinguish multiple legitimate transmitters and one spoofer in the MIMO-OFDM system, in which the proposed model can authenticate legitimate users and detect attackers by CSIs with higher performance compared to traditional hypothesis test-based methods.

To prevent spoofing and Sybil attacks, and ensure that authorized nodes in the IoT area have secure access, **(S26)** proposed a physical layer authentication mechanism based on CNN, using the association between instantaneous CSI and node position. Further, **(S16)** presented an adaptive neural network method to learn the features of the legitimate channel and perform spoofer detection. In addition, the study implemented the data-adaptive matrix and CNN frameworks for feature representation and classification. The results showed that the artificial neural network (ANN)-based method can significantly detect spoofing attackers, although the results are sensitive to the actual experimental scenario. Therefore, the adaptive neural network-based authenticator offers quick access authentication and improved security performance in wireless networks.

On the other hand, researchers in **(S29)** adopted three DL algorithms to implement physical layer authentication in industrial wireless sensor networks to meet low-latency requirements DNN, CNN, and the convolution preprocessing neural network (CPNN). Furthermore, they focused on the impact of different hidden layer numbers on authentication rates using the proposed method; the more hidden layers there are, the faster the neural network’s performance converges. Therefore, the authentication success rate increased as the number of hidden layers increased. Regardless, the method’s performance did not continue to improve and tended to stabilize once the number of hidden layers reached a certain number, due to the inherent characteristics of the specific wireless channels. The CNN and CPNN algorithms had good authentication performance and an ultra-short retraining time, whereas DNN had the best authenticating performance.

Using deep neural networks (DNN) for multiuser authentication has exceptional performance due to their excellent fitting and classification capabilities, but unfortunately, cannot perform well when the datasets are small. To this end **(S22)** proposed a multiuser authentication scheme that can identify multiple terminals simultaneously with low consumption by combining DNN with data augmentation methods. In **(S15)**, the authors considered deep-learning-based identification of near-field communication (NFC) tags by using RF fingerprinting to enhance security to prevent cloning attack. Various DNN models were adopted to extract RF features and achieve high identification accuracy. They considered three DNN models for RF fingerprinting of NFC tags a fully connected layer-based neural network (FNN), CNN, and RNN. In addition, they considered three popular machine-learning-based algorithms—logistic regression, random forest, and SVM. As a result, the performance of deep-learning-based algorithms surpassed that of conventional machine-learning-based algorithms. The study **(S31)** indicated that the inherent RF properties originating from the manufacturing process in a wireless device can be exploited as strong physical, unclonable functions for device authentication in asymmetric IoT networks without any additional hardware at the transmitters. Furthermore, a lightweight machine learning framework was designed to compensate for receiver non-idealities and simultaneously account for both data and channel variability. Since this is a nonlinear multidimensional classification problem, an ANN was employed as a learning engine.

On the other hand, **(S12)** utilised recurrent neural network’s (RNN) architecture in their study, in which they designed an intrusion detection technique that explores CSI to achieve secure and reliable performance in detecting adversarial intruders intelligently and precisely. Furthermore, to enhance detection accuracy and efficiency, they employed long short-term memory (LSTM) as an intelligent classifier to distinguish legitimate users from malicious intruders. The performance of the proposed LSTM-based detection technique was evaluated via simulations with variable channel conditions.

To evaluate the severity of adversarial attacks in wireless communications, **(S3)** considered an unauthorized transmitter trying to produce fake signals classified as authorized using deep learning. The researchers achieved this by recasting the problem as a reinforcement learning problem and proposed using policy gradient approaches to accomplish transmitter spoofing in a wireless network. They created a model that adds developed perturbations to the I/Q samples delivered by an adversarial transmitter to deceive the authenticator into identifying it as a legitimate transmitter. The proposed method considered neural network architecture for generators that employed LSTM networks; for discriminators, they used residual networks. The results demonstrated that it is possible to deceive a deep learning-based authenticator with high success rates even at low SNR.

However, because deep learning-based transmitter classification algorithms require training data to train the classifier, they are limited to known transmitters. As the attacker is unknown to the receiver, the receiver cannot acquire the attacker’s data to train these machine learning classifiers. Thus, these techniques are ineffective in the presence of an unknown attacker. A generative adversarial network (GAN)-based robust wireless transmitter identification scheme is proposed in **(S1)**; the authors offered a GAN-based wireless transmitter identification scheme to detect malicious attackers and classify trusted transmitters. Adversarial entities, such as rogue transmitters, might manipulate the signal and data when employing ML techniques for communication networks using different targeted data-generation techniques. In **(S2)**, the researchers demonstrated the use of GAN for the task of robust transmitter identification. The proposed GAN architecture has two primary components: a generative model that generates false data using given data distribution, and a discriminative model that estimates the probability that a sample came from the training data rather than the generative model. In the same context, **(S28)** developed an authentication model based on the physical layer in an adversarial environment. They employed the GAN model to detect the adversary, in which the discriminative model received either authentic samples from the training data or fake samples generated by the generative model. The generative model produced fake samples based on a function from random variable input and parameters. The discriminative model allocated a probability from zero to one based on whether the sample was fake or authentic. The discriminative model was trained to maximize the probability of allocating the correct label, whereas the generative model minimizes the same probability.

### 5.2. RQ2:What Are the Existing Physical Layer Authentication Techniques for Handling a Wireless Network’s Security Problems?

The various physical layer authentication techniques for a wireless network’s security problem offered by the studies included in this review are presented in this section. In addition, we addressed the use of radio frequency fingerprinting and channel-based techniques, as well as the research papers that have been written on each. Finally, Table 7 summarizes the PLA techniques and proposed solutions in the existing studies.

#### 5.2.1. Review of Selected PLA Technique-Based Radio Frequency Fingerprinting

Radio frequency fingerprinting is an effective and direct authentication technique that can enable ubiquitous computing to minimize authentication latency. However, the primary mission of authentication is to identify which device sent the unknown signal. To address this, **(S6)** used a one-against-one method that extended the SVM classifier to calculate the statistical features of many signals sent from one device. Furthermore, they utilised the Hilbert transform for feature extraction, which is considered the best of many straightforward methods to extract features from the original amplitude signal. The findings showed that using only the amplitude feature without any transforms, the classification accuracy was less than 40%, but the accuracy can reach 94% when using the Hilbert transform. In this regard, S5 proposed a radio frequency fingerprint extraction method based on fractional Fourier transform (FrFT) to distinguish different wireless network devices. To recover the useful low-rank matrix in the data and eliminate the sparse matrix of noise under certain conditions, they used robust principle component analysis (RPCA). RPCA is considered an excellent dimensionality reduction method that can achieve this goal. The proposed method results showed that when the SNR is 20 dB, the recognition rate was close to 100%.

In **(S11)**, the authors presented a radio ID verification-based IoT security approach using RF distinct native attribute (RF-DNA) fingerprints. The following are the eight feature selection techniques that were investigated in this study: dimensional reduction analysis (DRA), linear discriminant analysis (LDA), principal component analysis (PCA), neighbourhood component analysis (NCA), probability of error plus average correlation coefficient (POEACC), Bhattacharyya coefficient (BC), *t*-test, and relief-F. Selecting a model that is effectively adapted not only to verify the authorized radio’s ID but also to reject rogue radios masquerading as the authorized radio without having access to the rogue radios’ RFF during model building is an issue in ID verification. Setting a user/administrator-defined threshold is not required for ID verification and rogue radio rejection. Threshold-based techniques, on the other hand, require a compromise between the rate at which authorized radios’ IDs are verified vs. the rate at which rogue radios are rejected. As a result, increasing the ID verification rate necessitates sacrificing the rogue rejection rate. This study successfully demonstrated the performance result of true ID verification ≥ 90% at SNR ≥ 6 dB and the false rejection of rogue radio ≤10% at SNR ≥ 3. However, the dependency between verification and rejection rates leads to degraded model performance at lower SNR values.

Other studies used the physical characteristics of wireless devices to create unique authentication. In **(S7)** CNN combined with recurrence plots techniques was applied to represent the RF signal in 2D. The RF signal was sampled directly in the in-phase and Quadrature formats and then synchronized and normalized offline to extract the bursts of traffic associated with each payload. Moreover, **(S10)** even suggested a deep learning-based technology that used RF fingerprinting to recognize different devices accurately for secure wireless communication in a WLAN system. For device authentication, **(S14)** considered a method for Wi-SUN device authentication by extracting physical layer features, such as frequency deviation of FSK modulation and clock frequency offset, which differ slightly from device to device due to RF imperfections. Their proposed method successfully classified devices with 100% accuracy by observed physical layer fingerprints.

To solve the problem of identifying multiple of the identification of multiple wireless transmitters,. In **(S13)** used, the one-against-one SVM algorithm based on RF fingerprints through a short time Fourier transform to realize the classification and recognition of multiple wireless devices. They authors concluded that the correct recognition rate increased continuously rate increases continuously as the SNR increased. SNR increases. Further, in **(S1)**, a multi-classifier approach was presented to detect adversaries and classify trusted transmitters based on I/Q imbalance;, where I/Q imbalance is defined as the unique RF fingerprint of the different wireless transmitters. The classifier consists of multiple binary classifiers. Finally, the proposed method classifieds trusted transmitters with an average accuracy of 97.36% and detected adversaries with 99.98% accuracy.

Traditional cyberattack methods apply to IoT systems; therefore, security threat problems are significant obstacles to IoT development. Most IoT networks form a star network topology, and many devices connect to a central smart hub gateway that can act as the authenticator. The study **(S8)** established the RF authentication model by using RFF to effectively distinguish between authentic and rogue devices in the mobile IoT. In addition, they used methods in the authentication model—neighbourhood component analysis, support vector data description, and the swarm optimization algorithm. The model results showed that when the SNR exceeded 15 dB, the authentication success rate of this method exceeded 90%. In **(S4)**, a new access authentication method for IoT devices was proposed based on RFF technology. The identification process of wireless devices includes signal acquisition, using Hilbert transform to extract the signal feature, principal component analysis to reduce dimensionality, RFF for forming, and, finally, the KNN classifier for wireless device identification. The MITM attack is a common security problem that can damage industrial IoT. When an MITM attack was simulated in the study, the authors indicated that when the SNR was 10 dB, the attack detection probability was close to 100% and the authentication success rate was greater than 95%.

Cryptography based on a public key algorithm is not readily available for low-cost devices; for example, the processor of the NFC tag usually does not have enough computational power to process the public key algorithm. NFC security standards require public-key encryption/decryption to solve this issue, in terms of confidentiality and authenticity, and multiple attempts have been made to improve NFC’s authenticator. However, adversaries can exploit weaknesses in the stream cipher to read and modify memory blocks of an NFC tag. In NFC, a tag responds immediately to an NFC reader’s initiation, if the reader and the tag are nearby. In **(S15)**, the proposed identification scheme for NFC tags used RF fingerprinting, in which the goal was to enhance the security of NFC by preventing a cloning attack. Through evaluation, they confirmed that all considered deep learning structures operating in the proposed identification scheme achieved the highest accuracy for tag identification, with around 96%.

Traditional RFF techniques are becoming less prominent due to advanced manufacturing techniques, which have resulted in certain limitations. To address the issues of low reliability, reduced user capacity, diminishing distinguishability, and complications in the data processing of RFFs, **(S9)** proposed a novel injectable RFF scheme through electromagnetic metasurfaces. The authors created a metasurface method for radio frequency fingerprint injection (MeRFFI) to make small but detectable perturbations in the specific frequency band in which IoT devices communicate.

MeRFFI is designed for stationary IoT devices that communicate over wideband RF channels and is perfect for systems that require strong security, such as printers, health devices, wireless cameras, sensor network backhaul, and industrial monitoring systems. The method is incredibly energy efficient, as it can operate on zero power or a few hundred microjoules, depending on the application. The authors of **(S31)** developed a lightweight machine learning framework that compensates for receiver non-idealities and accounts for both data variability and channel variability simultaneously. The results demonstrated that the overhead of additional training iterations was useful for learning the channel conditions and variabilities, since each iteration has a distinct channel condition that the network learns to compensate for.

#### 5.2.2. Review of Selected PLA Technique-Based Channel Information

Multiple studies considered communication channel characteristics when building their authentication schemes. Channel characteristics generally provide richer information, although this requires more complexity to obtain a precise channel estimation. To address this, **(S19)** developed an approach for physical layer authentication by examining the most effective method for extracting channel differences from the channel matrix and employing several learning algorithms. In particular, the proposed approach achieved an average authentication accuracy of 77% across all the positions; when MIMO was used, performance was greatly improved across all positions in relation to SISO. Furthermore, **(S24)** deployed physical layer authentication approaches in cooperative communications, in which some trustworthy relay nodes assist a receiver in accurately authenticating a legitimate transmitter based on wireless channel characteristics. Furthermore, the authors proposed two feature selection models that can be used with statistical and ML-based classification techniques; they also considered equal gain combining (EGC) and all feature selection (AFS). When employing EGC, the statistical approach has a clear benefit, whereas AFS is best served by ML classification. This means that ML approaches benefit from more features than statistical approaches. In **(S25)**, researchers also used an ML algorithm to enhance physical layer security using channel features. The unique features of the physical layer channel are used to realize physical layer authentication. Mainly considered one-way authentication, the network authenticates the terminal and the terminal does network authentication, whereas mutual authentication is the combination of the two one-way authentications. In that study, the authors focused on 4G-LTE wireless communication technology like OFDM multi-carrier technology and MIMO multi-antenna transmission technology, taking the judgment condition of 4G mobile terminal cell reselection/cell handover into account.

Multiple studies proposed approaches for multiuser authentication when the traditional method cannot discriminate between multiusers simultaneously. For example, in **(S17)**, a multiuser authentication model was presented, distinguishing between multiple legitimate transmitters and one spoofer in the MIMO-OFDM system. They employed CNN and used the CSI of multiple transmitters as input and the corresponding tags as output to implement their model. As a result, the authentication rate was 90.5% when the number of iterations was eight; when the number of iterations was thirteen, the authentication rate was around 100%. As the number of iterations increased, the authentication rates of the test data also increased proportionally.

In **(S22)**, the authors proposed an approach combining DNNs with data augmentation techniques for multiuser authentication in open area test sites (OATS). Three data augmentation techniques were proposed and tested to enhance physical layer authentication datasets and effectively solve the problem of insufficient data volume. Furthermore, they tested the proposed authentication approach in two common industry IoT environments: OATS and an automotive factory. They also investigated how channel differences between these two representative environments influence the performance of the proposed approach. They concluded that the proposed approach provided faster and lighter-weight authentication and improved the authentication success rate. A new lightweight multiuser physical layer authentication mechanism was proposed in **(S27)**. The purpose was to prevent illegal nodes from imitating legitimate nodes to gain access to industrial wireless networks. The effectiveness of the proposed mechanism was verified by nodes in a real dynamic industrial scenario using the dataset collected by the National Institute of Standards and Technology (NIST).

In **(S29)**, a DL-based physical layer authentication approach was suggested that can differentiate between several industrial wireless sensor nodes at the same time while resisting spoofing. Without sacrificing communication resources, the proposed approach can improve the security of industrial wireless networks. Furthermore, three DL algorithms and the spatial diversity of wireless channels can discriminate the sensor nodes without imposing test thresholds and have more practical application values. Convolution preprocessing was employed to reduce the data dimension and extract the CSIs’ feature information, resulting in reduced training time and higher authentication accuracy. The authors also discovered that the cost function value declined as the number of iterations increased.

On the other hand, to enhance spoofing detection accuracy and efficiency, **(S12)** proposed an intrusion detection scheme that explores CSI to achieve secure and reliable performance. In addition, they employed LSTM as an intelligent classifier to distinguish legitimate users from adversaries. The proposed scheme’s results were accurate when evaluated via simulations with different channel conditions. In **(S18)**, researchers proposed an attack detection method based on channel differences. They implemented an automated labelling and learning technique for physical layer authentication to detect clone and Sybil attacks in edge computing industrial wireless networks. The CSI characteristics of a legitimate node are considered distinct from those of adversary nodes. Accordingly, an adversary node has multiple identities but only one physical device; when this adversary node launches Sybil attacks, the CSI from this node remains the same. They executed two attack scenarios to train the proposed method—clone and Sybil attacks. As a result, the detection accuracy rate in clone attacks reached 75%, whereas Sybil attack detection reached 84%. To prevent spoofing and Sybil attacks and ensure the secure access of legal nodes in the IoT area, **(S26)** proposed a physical layer authentication mechanism based on CNN, which utilises the strong coupling relationship between instantaneous CSI and node position. Therefore, CSI deep features are extracted as node identity to foreign unknown nodes within the pre-set area.

Any wireless communication system consists of access points and legitimate users, but there may be active eavesdroppers. To detect eavesdroppers who break into the system during the authentication phase, **(S30)** developed a framework for converting wireless signals into structured datasets that machine learning algorithms can use to detect active eavesdropping attacks at the physical layer.

Naturally, the performance of a physical layer authentication system is primarily affected by changing propagation and interference conditions. In **(S16)** the authors proposed an approach using an adaptive neural network (ANN) to detect changes in channel characteristics and determine whether an attack has occurred. To overcome the challenge of the physical layer, imperfect and noisy attribute measurements, the preprocessed data were to be authenticated with a wavelet-based noise filter. Then, they created a data-adaptive matrix for input to the classifier. This matrix consisted of a sequence of RSS vectors to capture the time-varying properties of the channel. Even if the data-adaptive channel matrix has improved the difference between legitimate and illegitimate channels, in a real communication environment, the RSS vectors are not necessarily the most practical features to use to differentiate a channel transmission from a spoofer.

On the other hand, **(S21)** presented the physical layer authentication algorithms by exploiting the channel states of the radio packets, which determine the test threshold based on reinforcement learning in dynamic wireless networks without knowing the complete channel parameters. Furthermoer, **(S28)** explained how the multiple subchannels concept might be used to achieve physical layer authentication. However, the authors also assumed that adversaries have the resources to change their antenna characteristics, transmitter RF path timing, and output power, and present reflectors between them and the receiver. For that, they implemented a discriminative model in the receiver, where adversaries were trained by a generative model that created authentic-looking CSI samples to defeat this scenario. For SNR ≥ 10 dB, the discriminator achieved 100% accuracy against the accidental authentication testing dataset; for SNR < 10 dB, the discriminator incorrectly classified legitimate samples. The proposed method can enhance the security of industrial wireless networks without sacrificing communication resources.

### 5.3. RQ3:What Are the Research Gaps in Wireless Network Security Based on Physical Layer Authentication Solutions?

Despite extensive research attention, physical layer authentication for wireless security is still struggling for practical deployment due to several challenges. We identified and discussed what we discovered during the systematic review to enhance the PLA research community.

#### 5.3.1. Main Challenges

- The first challenge is that the wireless network environment changes dynamically over time [83,84,100]. This is because wireless channel conditions vary significantly according to configuration, and the shape of the RF signal changes according to variations in wireless channel conditions, which can affect transmitter identification.
- Differences in the relevant hardware feature devices are relatively small. In addition, noise and interference further distort feature detection, reducing the accuracy of estimating them for authentication purposes.
- Since wireless communication transmission exists in a random fading environment, imperfect estimation and incomplete measurement of wireless signals are unavoidable [84], resulting in unpredictably varying authentication systems.
- The rapid improvement in operational wireless infrastructure supported the dramatically increased traffic [100]. As a result, the complexity of wireless networks will grow, and wireless device users will have to switch between multiple base stations or access points more frequently, resulting in frequent authentication handovers.

#### 5.3.2. Open Issues and Future Trends

This section addresses key issues that have not yet been extensively investigated and future trend solutions.

**Figure 9:**
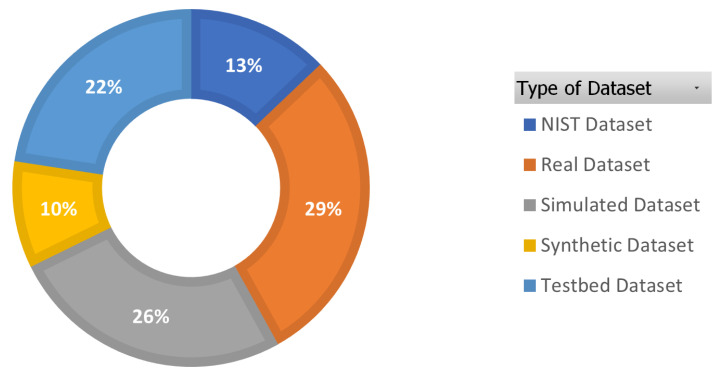
Dataset types used in selected papers.

**Datasets Issues:** Most studies’ experiments faced a lack of sufficient data, which affected the results of the proposed classifiers. When **(S14)** faced this issue, they implemented their experiment with a small number of wireless smart utility devices. In **(S22)**, the authors used the data augmentation technique to regenerate datasets from existing datasets by computing operations, which is an efficient way to expand limited training datasets to build an accurate authentication model for ML and DL algorithms. However, deep learning approaches require many training samples to achieve high accuracy. Recently, few studies have tried to utilize a generative model to overcome the lack of training samples. Both **(S2)** and **(S15)** used the GAN framework as a generative model, which allows realistic generation of samples from a particular distribution that can then be used to train a discriminator to distinguish real samples from those created by the generator. Figure 9 represents the percentage of dataset types in the reviewed papers.

**Environment Issues:** The shape of the RF signal can change according to variations in wireless channel conditions, which can affect tag identification. Furthermore, a channel-based PLA and the transmitter’s location may also significantly influence authentication performance because the channel depends on a party of transceivers. Consequently, extra effort should be exerted for PLA under a dynamic channel environment. To solve the problem of varying channels dynamically, RF signal data must be collected under different channel conditions so that ML and DL models can cope with unstable channel conditions. On this topic **(S29)** and **(S31)** concluded that as the number of transmitters increases, the authentication error rate increases even if distances decline. In addition, noise, attenuation in the communication medium, interference, Doppler shift, and fading affect dynamic channel variation in short-range communication. Because of time and space constraints, most reviewed papers only discussed recognition methods based on specific channel conditions. As a result, the real wireless channel effect is not entirely reflected. Therefore, future studies should use a functional model to describe the unique physical layer differences of devices. Furthermore, the channel influence must be separated from the function model so that this technology can be used in scenarios in which the channels are dynamic. In the same context, considering the mobility of devices in the IoT and the characteristics of dense access networks of terminal devices, **(S16)**, **(S25)**, and **(S27)** pointed out that PLA models for moving devices have been a more challenging issue than authenticating static devices. In the future, further studies can be conducted on physical layer authentication in advanced wireless communication technology terminals and IoT devices with mobility and high-speed environment scenarios.

**ML/DL Approache Issues:** Machine learning has recently received attention in the communication domain because of successful deep learning approaches in computer vision, automatic speech recognition, and natural language processing. However, unlike computer vision, in which the dataset is generally represented as pixel values, communication system design is based on real-world channel conditions and RF signals [101], which are unpredictable and varied. Moreover, we have noticed that current authentication DL models are mainly trained offline. Therefore, it is imperative to design models for specific or general scenarios that dynamically adapt to varying channels. For example, in **(S9)**, the authors referred to CNN as a robust technique in a static environment, but if the receiver changed orientation or had to be moved from one place to another, the system would have to be retrained. Most artificial intelligence algorithms for wireless communication network physical layers are still in the simulation stage. Wang et al. [101] pointed out how to overcome ML and DL technique-related issues; researchers must be assisted in training their models on common measurable data and accurately assessing the performance of different algorithms, and authentic datasets from real communication systems or prototype platforms in real physical surroundings must be made available to all researchers.

Furthermore, the presence of adversaries makes it even more challenging to learn and characterize RF signals because of the unreliability of the underlying data. In **(S2)**, the authors pointed out that most traditional ML techniques are susceptible to malicious attacks. The susceptibility increases once the attacker knows the features used by the learning algorithm. The attacker becomes smart enough to mislead the learning process. With this knowledge, adversaries can use a generative model to generate signals to spoof the transmission of known transmitters. This renders moot the traditional learning algorithms in wireless channels. Comparing machine learning and deep learning algorithms in the context of PLA remains an open issue for further research. However, with the potential of improved data augmentation techniques, we recommend delving deeper into the data to explore hidden features that can help detection and authentication models perform even better.

## 6. Conclusions

This paper presented an SLR of physical layer authentication used to secure wireless communication and networks. We selected several well-known databases as reliable electronic sources, such as IEEE Xplore, ACM Digital Library, Science Direct, Springer, and Hindawi. First, 248 papers published between 2015 and 2022 were selected. Then, according to the inclusion/exclusion criteria, 31 of the 248 papers were selected to analyse and exploit the appropriate data. We arranged this paper based on three research questions: RQ1, RQ2, and RQ3 were answered by analysing the selected papers.

Regarding RQ1, the selected studies determined the algorithm types, evaluation types, features, and tools used. In the overview of papers, it was observed that 45% of selected papers were machine learning-based, and 55% were deep learning-based. In addition, a comparison of evaluation types showed that 69% applied a simulation environment to appraise the PLA in different wireless network environments. Based on RQ2, the PLA techniques in the studied papers were discussed; the comparison of the PLA techniques category indicated that 52% of the studied papers employed RF characteristics, and 48% used channel characteristics to implement PLA systems. Finally, according to RQ3, to develop more efficient PLA approaches in the future, we have described the open challenges, issues, and future trends of PLA in wireless networks and communication.

As a result of the rapid development of wireless communications and increasing security threats, the physical layer security of wireless networks is becoming increasingly important. We expect these findings to aid other researchers in developing PLA systems more effectively. Although earlier research has achieved promising results, some significant issues should be investigated further in future studies. Furthermore, research into ML and DL approaches to physical layer security of wireless networks is still in its early stages and deserves further investigation.

## Figures and Tables

**Table 1:** Comparison of related works.

Study	Publication Year	Publisher	Topics	Review Type	Covers ML	Environment	Focused Issues
[2]	2018	IEEE	PLS	Survey	No	Wireless Network	The optimization and design in PLS
[3]	2018	IEEE	PLS	Survey	No	5G Wireless Networks	PLS techniques provide for 5G wireless networks
[4]	2016	IEEE	PLS	Survey	No	Wireless Network	The PLS techniques, and challenges
[10]	2021	Springer	PLS/PLA	Survey	Yes	Wireless Network	PLS techniques based on machine learning
[25]	2013	IEEE	PLS	Survey	No	Cognitive Radio Networks	PLS in cognitive radio networks
[26]	2019	IEEE	PLS	Survey	No	5G Wireless Networks	The PLS on enabling technologies for 5G
[27]	2021	IEEE	PLS	Survey	No	IoT Communications	PLS techniques in the IoT communication protocols
[28]	2020	IEEE	PLA	Survey	Yes	Wireless Network	PLA techniques and challenges
[29]	2021	IEEE	PLA	Survey	No	Wireless Network	PLA techniques
[30]	2020	IEEE	PLA	Survey	Yes	5G Wireless Networks and IoT Communications	PLA schemes using machine learning for the 5G-based IoT
[31]	2022	IEEE	PLS	Survey	No	Wireless Network	Physical layer security challenges

**Table 2:** Research questions and motivations.

Research Questions	Motivations
RQ1: What machine learning approaches are applied in physical layer authentication to secure the wireless network?	To assess whether machine learning approaches in physical layer authentication models enhance the performance of wireless network security.
RQ2: What are the existing physical layer authentication techniques for handling a wireless network’s security problems?	To identify what techniques have been used in physical layer authentication.
RQ3: What are the existing key challenges, open issues, and future trends in wireless network security based on physical layer authentication solutions?	To identify gaps in the physical layer authentication literature on wireless network security and to suggest directions for future research.

**Table 3:** Inclusion and exclusion criteria to select the primary studies.

Inclusion Criteria	Exclusion Criteria
Studies related to physical layer authentication and wireless network or IoT security.	Studies written in languages other than English.
Studies related to transmitter classification and using deep learning techniques or machine learning techniques.	Studies that are reviews or surveys without findings.
Studies published from January 2015 to January 2022.	Studies without justifiable research contributions.

**Table 4:** Studies that met the quality evaluation criteria.

Study Number	Title	Authors	Year	Publisher	Type
S1	Radio Frequency Fingerprint Based Wireless Transmitter Identification Against Malicious Attacker: An Adversarial Learning Approach	Hao Han et al. [70]	2020	IEEE	Conference
S2	RFAL: Adversarial Learning for RF Transmitter Identification and Classification	Debashri Roy et al. [71]	2020	IEEE	Journal
S3	Penetrating RF Fingerprinting-based Authentication with a Generative Adversarial Attack	Samurdhi Karunaratne et al. [72]	2021	IEEE	Conference
S4	New Security Mechanisms of High-Reliability IoT Communication Based on Radio Frequency Fingerprint	Qiao Tian et al. [73]	2019	IEEE	Journal
S5	Improving Wireless Network Security Based On Radio Fingerprinting	Yun Lin et al. [74]	2019	IEEE	Conference
S6	Identification and authentication for wireless transmission security based on RF-DNA fingerprint	Xueli Wang et al. [75]	2019	Springer	Journal
S7	Physical layer authentication of Internet of Things wireless devices using convolutional neural networks and recurrence plots	Gianmarco Baldini et al. [76]	2018	Wiley	Journal
S8	An Identity Authentication Method of a MIoT Device Based on Radio Frequency (RF) Fingerprint Technology	Qiao Tian et al. [77]	2020	MDPI	Journal
S9	Injecting Reliable Radio Frequency Fingerprints Using Metasurface for the Internet of Things	Sekhar Rajendran et al. [78]	2021	IEEE	Journal
S10	Securing Wireless Communication Using RF Fingerprinting	Nur Sabryna Aminuddin et al. [79]	2021	IEEE	Conference
S11	Radio Identity Verification-Based IoT Security Using RF-DNA Fingerprints and SVM	Donald Reising et al. [80]	2021	IEEE	Journal
S12	A LSTM-Based Channel Fingerprinting Method for Intrusion Detection	Ting Ma et al. [7]	2021	IEEE	Conference
S13	Identification of Wireless Transceiver Devices Using Radio Frequency (RF) Fingerprinting Based on STFT Analysis to Enhance Authentication Security	Songlin Chen et al. [81]	2017	IEEE	Conference
S14	Wi-SUN Device Authentication using Physical Layer Fingerprint	Mi-Kyung Oh et al. [82]	2021	IEEE	Conference
S15	Deep-Learning-Aided RF Fingerprinting for NFC Security	Woongsup Lee et al. [83]	2021	IEEE	Journal
S16	A Learning Approach for Physical Layer Authentication Using Adaptive Neural Network	XIAOYING QIU et al. [84]	2020	IEEE	Journal
S17	A Novel Physical Layer Authentication Method with Convolutional Neural Network	Runfa Liao QIU et al. [85]	2019	IEEE	Conference
S18	Automated Labeling and Learning for Physical Layer Authentication Against Clone Node and Sybil Attacks in Industrial Wireless Edge Networks	Songlin Chen et al. [86]	2021	IEEE	Journal
S19	Threshold-Free Physical Layer Authentication Based on Machine Learning for Industrial Wireless CPS	Fei Pan et al. [87]	2019	IEEE	Journal
S20	Physical layer Channel Authentication for 5G via Machine Learning Algorithm	Songlin Chen et al. [88]	2018	Hindawi	Journal
S21	PHY-Layer Spoofing Detection With Reinforcement Learning in Wireless Networks	Liang Xiao et al. [89]	2016	IEEE	Journal
S22	Multiuser Physical Layer Authentication in Internet of Things with Data Augmentation	Run-Fa Liao et al. [90]	2019	IEEE	Journal
S23	Machine Learning-based Physical Layer Authentication using Neighborhood Component Analysis in MIMO Wireless Communications	Jiseok Yoon et al. [91]	2019	IEEE	Conference
S24	Physical Layer Authentication with Cooperative Wireless Communications and Machine Learning	Linda Senigagliesi et al. [92]	2021	IEEE	Conference
S25	Physical Layer Authentication Algorithm Based on SVM	Chuping Dai et al. [93]	2016	IEEE	Conference
S26	A Physical Layer Authentication Mechanism for IoT Devices	Xinglu Li et al. [8]	2021	IEEE	Journal
S27	Multiuser physical layer security mechanism in the wireless communication system of the IIOT	Ruizhong Du et al. [94]	2022	Science Direct	Journal
S28	Physical layer Authentication Using Channel State Information and Machine Learning	Ken St. Germain, Frank Kragh [33]	2020	IEEE	Conference
S29	Deep-Learning-Based Physical Layer Authentication for Industrial Wireless Sensor Networks	Run-Fa Liao et al. [53]	2019	MDPI	Journal
S30	Physical Layer Security: Detection of Active Eavesdropping Attacks by Support Vector Machines	TIEP M. HOANG et al. [95]	2021	IEEE	Journal
S31	RF-PUF: Enhancing IoT Security through Authentication of Wireless Nodes using In-situ Machine Learning	Baibhab Chatterjee et al. [96]	2018	IEEE	Journal

**Table 5:** Quality assessment of the selected studies.

Study Number	QA1	QA2	QA3	QA4	QA5	QA6	QA7	QA8	QA9	QA10	Total
S1	1	0.5	1	1	1	1	1	1	1	1	9.5
S2	1	1	0.5	1	1	1	1	1	1	1	9.5
S3	0.5	0.25	0.25	0.75	0.5	0.75	0.25	0.5	0.75	1	5.5
S4	0.75	1	1	1	0.5	1	0.25	0.5	1	1	8.25
S5	0.5	0.5	0.25	0.25	1	0.75	0.25	0.5	0.5	1	5.5
S6	1	0.75	0.75	1	1	1	0.5	1	1	1	9.5
S7	1	0	0.5	0.25	1	1	0.75	0.75	0.5	0.5	6
S8	1	1	0.25	1	1	1	0.5	1	1	0.75	8.25
S9	1	1	1	1	0.25	1	0.5	1	1	1	8.5
S10	1	1	1	1	1	1	0.75	1	0.75	1	9.5
S11	1	1	1	1	1	1	0.75	1	1	1	9.75
S12	1	0.75	1	1	0.75	0.5	0.25	0.5	0.5	0.75	7
S13	0.5	0.25	0	1	1	1	0.5	0.5	0.5	1	6
S14	0.75	0.5	0.75	1	0.25	1	0.25	0.5	0.5	1	6.5
S15	1	1	1	1	1	1	1	1	1	1	10
S16	1	1	1	1	1	1	0.5	1	1	1	9.5
S17	0.75	0	0.75	1	1	1	0.25	0.5	0.75	1	7
S18	1	1	1	1	1	1	0.5	1	1	1	9.5
S19	1	0.75	1	1	1	1	1	1	1	0.75	9.5
S20	1	0.75	1	1	1	1	0	1	0.75	1	8.5
S21	1	1	0.5	1	1	0.25	0.25	0.25	1	1	7.25
S22	1	1	0.5	1	1	0.75	0.75	0.75	1	1	8.75
S23	0.75	0.25	0.5	1	0.25	1	0.25	0.5	0.5	0.75	5.75
S24	0.75	0.25	0.75	1	0.25	1	0.25	0.75	0.75	0.5	6.25
S25	0.5	0.25	0.75	1	1	1	0.25	0.75	0.25	0.5	6.25
S26	1	0.5	1	1	1	1	0.25	1	1	1	8.75
S27	1	0.5	0.5	0	1	1	0.75	1	1	1	7.75
S28	1	1	0.75	1	1	1	0.75	1	1	1	9.5
S29	1	1	1	1	1	1	0.25	1	1	1	9.25
S30	1	1	1	1	1	1	0.5	1	1	1	9.75
S31	1	1	1	1	1	1	1	1	1	1	10

**Table 6:** Information of the reviewed PLA based machine learning approaches.

Study Number	Approaches	Algorithms	Evaluation Technique	Features	Performance Metrics	Tools/Platforms
S1	DL	GAN-CNN	Simulated	In phase and Quadrature Data	Confusion Matrix of classification accuracy	Python (TensorFlow)
S2	DL	CNN-DNN-RNN	Simulated/Real test	In phase and Quadrature Data	ROC Curve and Confusion Matrix of classification accuracy	Python (Keras/ TensorFlow)-GNURadio
S3	DL	RNN	Simulated/Real test	In phase and Quadrature Data	Fooling Rate for different levels of SNR	Python (Keras/pyadi-iio)
S4	ML	KNN	Simulated	Amplitude Envelope (using Hilbert transformation)	Confusion Matrix of classification accuracy	MatLab
S5	ML	KNN	Simulated	Signal information in the time and the frequency domain (using Fractional Fourier Transform)	Confusion Matrix of classification accuracy	MatLab
S6	ML	SVM	Simulated	Phase-Frequency-Amplitude (using Hilbert transformation)	Classification Accuracy	MatLab
S7	DL	CNN	Simulated	In phase and Quadrature Data	Confusion Matrix of classification accuracy	Recurrence Plots (visualization tool)
S8	ML	SVM	Simulated	Amplitude Envelope (using Hilbert transformation)	Authentication/ Detection success rate	MatLab
S9	DL	CNN	Real test	Channel State Information	ROC Curve and Classification Accuracy	Not mentioned
S10	DL	CNN	Simulated	In phase and Quadrature Data	Confusion Matrix of classification accuracy	MatLab
S11	ML	SVM	Simulated	In phase and Quadrature Data	Vérification Rate	Not mentioned
S12	DL	RNN	Simulated	Channel State Information	Confusion Matrix of detection accuracy	Not mentioned
S13	ML	SVM	Simulated	Time-Frequency (using The Short Time Fourier Transform)	Recognition Rate	MatLab–GNURadio
S14	ML	KNN	Real test	Frequency (using Fast Fourier Transform)	Confusion Matrix of classification accuracy	MatLab
S15	DL	CNN-DNN-RNN	Real test	RF characteristics of an NFC tag	ROC Curve and Confusion Matrix of classification accuracy	GNURadio
S16	DL	CNN	Real test	Received Signal Strength Amplitudes	Detection Rate	USRP
S17	DL	CNN	Simulated	Channel State Information	Authentication Accuracy	Not mentioned
S18	ML	SVM	Simulated/Real test	Channel State Information	ROC Curve-Authentication Accuracy	USRP
S19	ML	SVM-DT-KNN	Simulated/Real test	Channel State Information	Authentication Accuracy	USRP
S20	ML	SVM-DT	Real test	Channel State Information	Detection Rate	MatLab–USRP
S21	ML	Reinforcement	Real test	Channel State Information	Detection Rate	USRP
S22	DL	DNN-CNN-CPNN	Simulated	Channel Impulse Response	Authentication Accuracy	Not mentioned
S23	ML	SVM-NCA	Simulated	Channel Impulse Response	ROC Curve of Authentication Accuracy	Not mentioned
S24	ML	KNN	Simulated	Channel Frequency Responses	Confusion Matrix of classification accuracy	Not mentioned
S25	ML	SVM	Simulated	Channel information and responses	Classification Accuracy	MatLab-LTE Simulator-QUALNET
S26	DL	CNN	Simulated	Channel State Information	Authentication Accuracy	Quasi-Deterministic Radio Channel Generator (QuaDRiGa)
S27	ML	SVM	Simulated	Channel Frequency Responses (using Fast Fourier Transform)	Authentication Accuracy	MatLab
S28	DL	GAN	Simulated	Channel State Information	Confusion Matrix of classification accuracy	Python (Keras/ TensorFlow)
S29	DL	DNN-CNN-CPNN	Simulated/ Real test	Channel State Information	Authentication Accuracy	USRP
S30	ML	SVM	Simulated	Channel State Information	ROC Curve of classification accuracy	Python
S31	DL	ANN	Simulated	In phase and Quadrature (Frequency-Amplitude)	Classification Accuracy	MatLab-GNURadio

**Table 7:** Authentication techniques, proposed solution of existing studies on PLA.

Study Number	Authentication Techniques	Proposed Solutions
S1	RFF	Radio frequency fingerprint classifier consists of multiple discriminators to both detect malicious attackers and classify trusted transmitters.
S2	RFF	Radio frequency adversarial learning model generates fake signals and distinguishes trusted transmitters from rogue ones.
S3	RFF	Deep learning-based classifier to evaluate the feasibility of using physical layer authentication by introducing an algorithm that adds learned perturbations transmitted by an adversarial transmitter to fool the authenticator and classifying it as an authorized transmitter.
S4	RFF	Radio frequency fingerprint security mechanism to avoid the man-in-the-middle attack in industrial IoT scenario.
S5	RFF	Method based on radio frequency fingerprint to enhance wireless network security and distinguish different wireless network devices.
S6	RFF	Technology based on radio frequency fingerprint to find the difference among devices and identify them.
S7	RFF	Deep learning-based approach for the authentication of IoT wireless devices with the same model.
S8	RFF	Radio frequency fingerprint authentication model to solve identity authentication problems in the mobile IoT.
S9	RFF	Inject a designed radio frequency fingerprint into the wireless physical layer to increase the security of a stationary IoT device with minimal overhead.
S10	RFF	Radio frequency fingerprint models utilize raw baseband In-phase and Quadrature samples to identify the transmitting radio.
S11	RFF	Physical layer IoT authentication approach based on radio frequency fingerprint to successfully authorize identity (ID) verification and rejection of all rogue radio ID spoofing attacks.
S12	RFF	Implement an intrusion detection scheme to determine whether a spoofing attack happens.
S13	RFF	Radio frequency fingerprint method to identify the same wireless transceiver module.
S14	RFF	Radio frequency fingerprint classifier based on machine learning to identifies the authorized wireless smart utility network devices.
S15	RFF	Near field communication tags identification method based on deep learning and radio frequency fingerprint to enhance the security by preventing the cloning attack.
S16	RSS	Adaptive neural network authentication process to improve from attack detection and leading to effective physical layer security.
S17	CSI	A multi-user authentication system security to detecting spoofing attacks in wireless networks.
S18	CSI	Automated labeling and learning method for physical layer authentication where detect clone and Sybil attacks in edge computing industrial wireless network.
S19	CSI	Physical layer authentication approach based on machine learning algorithms in industrial wireless cyber-physical systems.
S20	CSI	Physical layer authentication method-based machine learning and channel features for the 5G wireless communication security by determining whether the received packets are from a legitimate transmitter or a counterfeiter.
S21	RSSI	PLA spoofing detection schemes based on ML in wireless networks.
S22	CIR	Deep learning based physical layer authentication framework to enhance the security of industrial wireless sensor networks.
S23	CIR	Machine learning based physical layer authentication scheme in the multi-input and multi-output wireless communication environment.
S24	CFR	Physical layer authentication approaches based on statistical and machine learning techniques.
S25	CSI	Wireless physical layer channel authentication classifier combined with machine learning algorithm.
S26	CSI	Physical layer authentication mechanism based on deep learning and wireless channel fingerprints to distinguish sending nodes in different locations.
S27	CFR	Physical layer authentication scheme for multiuser to improve the accuracy of authentication in dynamic industrial scenarios.
S28	CSI	Physical layer authentication method uses an adversarial neural network and measured multiple-input multiple-output communications channel information to decide on whether to authenticate a particular device.
S29	CSI	Deep learning based physical layer authentication methods to enhance the security of industrial sensor networks by utilizing the spatial diversity of wireless channels.
S30	CSI	Machine learning classifiers are considered to detect the eavesdropper who breaks into the system during the authentication phase.
S31	RFF	Radio frequency Physical unclonable function method where allows real-time authentication of wireless nodes that not require any additional circuitry for generation or feature extraction.

## Abbreviations

The following abbreviations are used in this manuscript:

PLS	Physical layer security
PLA	Physical layer authentication
RFF	Radiofrequency Fingerprint
CSI	Channel State Information
RSS	Receive Signal Strength
CIR	Channel Impulse Response
